# Subjective Psychological Well-Being in Families with Blind Children: How Can We Improve It?

**DOI:** 10.3389/fpsyg.2016.00487

**Published:** 2016-04-05

**Authors:** Juan J. Sola-Carmona, Remedios Lopez-Liria, David Padilla-Gongora, María T. Daza, Jose M. Aguilar-Parra

**Affiliations:** ^1^National Organization of Spanish BlindAlmería, Spain; ^2^Department of Nursing Science, Physiotherapy and Medicine, University of AlmeríaAlmería, Spain; ^3^Department of Psychology, University of AlmeríaAlmería, Spain

**Keywords:** blind children, anxiety, well-being, job satisfaction, family satisfaction

## Abstract

The aim of this work was to examine family well-being in a sample of Spanish families with blind children. Sixty-one participants reported their perceived economic status, the level of job satisfaction, and state-anxiety symptoms. The participants of our study scored higher on state-anxiety and lower on material well-being than the normative sample, although these differences did not reach statistical significance. They also scored higher on job satisfaction and family satisfaction than the general population. A negative correlation was found between state-anxiety and material well-being (*r* = - 0.62, *p* = 0.001) and between state-anxiety and family satisfaction (*r* = - 0.57, *p* = 0.001). A positive correlation was found between material well-being and job satisfaction (*r* = 0.40, *p* = 0.001), and between material well-being and family satisfaction (*r* = 0.41, *p* = 0.001). Higher levels of material well-being, job satisfaction, and family satisfaction were associated with lower levels of anxiety in these families. However, no statistically significant correlation was found between family satisfaction and job satisfaction. Our results suggest that the family experience of having a disabled child is evolving, and this implies achieving greater job and family satisfaction than the normative samples, although anxiety scores continue to be higher and material well-being scores remain lower. On the whole, our results confirm that it is necessary to provide these families with more economic resources, which would have a positive impact on their subjective psychological well-being, decreasing their state-anxiety, and increasing their satisfaction with life.

## Introduction

The repercussions of a disability on children and their parents are currently the subject of social and scientific interest. Some previous studies describing how the birth of a disabled child affects family coexistence have found negative consequences as a result of paying too much attention to the child, for example, the high social and emotional cost, which can turn into a powerful source of anxiety and which has an impact on subjective well-being (Diener and Lucas, 2000; Eisenhower et al., 2009; Hatton et al., 2010; Martorell et al., 2011).

According to the tridimensional model of Diener and Lucas (2000), subjective psychological well-being is made up of positive affect (a subjective component of emotions like joy, pleasure, and euphoria), negative affect (fear, stress, and sadness), and satisfaction with life, understood as the subjective appraisal of well-being at work, in the family, and with regard to economic income and leisure.

Other authors, like Emerson et al. (2010), have defended that, if the fact that these families are more exposed to socioeconomic disadvantages is not taken into account, the relation between the disabled child and the parents’ lack of subjective well-being may be overestimated (Emerson and Hatton, 2008). Thus, for example, according to Leyser and Heinze (2001), long-distance trips to provide special services are one of the main priorities of parents with visually impaired children. This prevents the children from taking part in other activities of their community, reducing their integration. At the same time, it implies a great overload in terms of economy and dedication, affecting families’ work, leisure time, and social relations. In a study conducted in Spain, Calvo and González (2004) observed that having a child with a visual impairment requires the mobilization of resources to cover the child’s needs. This implies an increase of expenses and/or a relevant decrease of family income. These circumstances worsen when these families already have a low socioeconomic level. Thus, it has been observed that the mother frequently quits her job, reduces her workday schedule, or else the family is forced to hire support personnel so that the visually impaired child will receive specialized educational or therapeutic services, which are usually offered by the public administration, organizations for disabled people, or private groups (Calvo and González, 2004; Montes and Halterman, 2008).

This situation may also produce exhaustion and tension in the couple relation due to arguments and less consensus or mutual understanding among family members when making decisions. Changes in the family dynamics have also been reported, as the members may feel unsure and doubtful about their capacity to care for the child with disabilities (Berg et al., 2007). All of this causes symptoms of depression and anxiety and poor quality of life (Brehaut et al., 2009; Hutchinson et al., 2009; Kuhlthau et al., 2010). It would therefore be interesting to have deeper knowledge of the relation between the key factors that contribute to the subjective well-being of blind children’s parents. In others words, would be interesting to examine the relationships between the three components of the subjective psychological well-being model (Diener and Lucas, 2000) in the parents of blind children. As stated by other authors, the parents of children with some type of disability act as mediators between their children and society (Ojeda and Mateos, 2006). They are the ones who can best determine their children’s needs and well-being (Dempsey and Keen, 2008) and, ultimately, the ones who can promote their development (Calvo and González, 2004). However, there are few studies on the subjective psychological well-being of parents with disabled children, and particularly in the case of visual impairment (Leyser and Heinze, 2001; Dunst and Dempsey, 2007; Dempsey and Keen, 2008; Dempsey et al., 2009; Feniger-Schaal and Oppenheim, 2013; ?; Holbrook, 2015).

Accordingly, our first goal consisted of measuring the three components of subjective psychological well-being in a Spanish sample of parents of blind children, and determining possible significant differences compared with the scores of the normal population. Specifically, to address the component of positive affect, we used a measure of family satisfaction (Barraca Mairal and Yarto-López Elizalde, 2003); for negative affect, we used a measure of state-anxiety (Spielberger et al., 1983; Spielberger, 1988; Spielberger and Sydeman, 1994); and for the component of satisfaction with life, a measure of material well-being (Sánchez-Cánovas, 2007), and one of job satisfaction (Sánchez-Cánovas, 2007). The second goal consisted of studying the relation between these components in the parents of blind children.

Previous studies of families with visually impaired children led us to hypothesize that the level of state-anxiety in blind children’s parents would be higher than that of the general population. In contrast, material well-being, job satisfaction, and family satisfaction would be lower in families with blind children.

## Materials and Methods

### Participants

The total population of this investigation comprised 95 parents from the province of Almería (southern Spain), whose children fulfilled the ophthalmic requirements for affiliation in the National Organization of Spanish Blind (ONCE).

Parents who took part in this study were chosen according to the following selection criteria: perfect knowledge of Spanish language, efficient reading comprehension, having children affiliated with the ONCE and who were also assisted by the specialized orientation staff for blind and visually disabled people of this province (which has a total population of about 700.000 inhabitants).

The exclusion criteria were: parents who refused to give their consent to participate and parents whose children did not receive assistance directly from the specialized orientation team for blind people in Almería.

The initially selected sample consisted of 81 fathers or mothers (not couples; they all belonged to different families), of whom 61 completed the questionnaires. In total, 34 participants were excluded (20 participants refused to take part in the study and 14 were parents of children who were not attended to by the orientation team).

The final sample comprised 28 fathers (45.90%) and 33 mothers (54.10%), aged between 25 and 58 years old (mean age 41.52 ± 5.90 years) of 61blind children. Of these parents, 82% were married in first nuptials, and 30% had one child (the one with the visual disability). Only 39.30% had a higher educational degree, and 60.70% were working, that is, receiving work wages.

Concerning the children of these families, 13.10% were totally blind, 34.40% were visually impaired, and 52.50% were visually impaired and also had other physical disabilities as well as intellectual disability. Their mean age was 9.16 ± 4.90 years, and 62.30% were male.

### Procedure

This is a descriptive, cross-sectional study in which data were collected through questionnaires. First, the institutional review board at the Psychology Department in the University of Almería reviewed this project and granted permission to implement it. Members of the orientation staff of the ONCE contacted the selected families personally and the parents received a study description with a consent form to participate in this study. After explaining the aims of the investigation to the parents, 81 questionnaires with instructions on how to complete them were sent by postal mail to those who consented to participate in the study. The parents of the contacted families filled in the questionnaires independently, and 61 questionnaires were returned to the researchers by postal mail.

### Instruments

To select the instruments for data collection, we drew on the model of subjective psychological well-being proposed by Diener and Lucas (2000), and on previous studies like that of Barraca Mairal and Yarto-López Elizalde (2003), which indicated important factors related to the construct of subjective psychological well-being, such as state-anxiety, economic well-being (both material and job satisfaction), and family satisfaction (understood as the cognitive and affective appraisal of verbal and physical interactions with the family members).

(1) The State-Anxiety Subscale of the Spanish version of the State-Trait Anxiety Inventory-STAI (Spielberger et al., 1983; Spielberger, 1988; Spielberger and Sydeman, 1994). This provides a measure of state-anxiety, defined as a transitory condition or emotional state, characterized by subjective feelings of tension and apprehension as well as hyperactivity of the autonomic nervous system, which may change over time and fluctuate intensively. The subscale has 20 items, with a minimum score of 0 and a maximum score of 60, with higher scores indicating more anxiety. Items are rated from 0 to 3 points depending on the degree of anxiety experienced. Ten items are scored positively, and ten are inversely scored. In the Spanish standardized version of the scale, Cronbach’s alpha reliability ranged between 0.90 and 0.93. Using the split-half method, reliability was 0.94. In our study, Cronbach’s alpha was 0.93. These data are similar to the original studies of this test (Spielberger and Sydeman, 1994). In the present study, the data from our sample were compared with those obtained with the same scale in the normative sample of adolescents and normal adults, aged between 16 and 62 years old (45% male and 55% female). Concerning gender, the standardization sample included 295 males with a mean State-anxiety score of 20.54 (*SD* = 10.56) and 365 females, with a mean State-anxiety score of 23.30 (*SD* = 11.93).
(2) Material Well-Being Subscale of the Spanish “Escala de Bienestar Psicológico” (EBP; in English, the Psychological Well-Being Scale; Sánchez-Cánovas, 2007), which measures the subjective perception of the respondent’s economic situation (income and quantifiable material possessions). It has 10 items on which the frequency or degree of agreement with the statement is rated on Likert-type scales ranging from 1 to 5, with higher scores indicating higher subjective perception of material well-being (maximum score is 50). Cronbach’s alpha reliability was 0.91. The concurrent validity of this subscale with the Oxford Happiness Questionnaire was 0.53 (Argyle et al., 1989; Hills and Argyle, 2002). In our study, Cronbach’s alpha was 0.88. The standardization of this test was conducted in a sample made up of 1885 people from the general population. The mean score of the sample of the test was 33.48 (*SD* = 8.90).
(3) Job Satisfaction Subscale of the Spanish “Escala de Bienestar Psicológico” (EBP; in English, the Psychological Well-Being Scale; Sánchez-Cánovas, 2007). This scale provides a measure of the level of job satisfaction (positive emotional response to work). It has 10 items that are rated on Likert-type scales ranging from 1 to 5, with higher scores indicating a greater job satisfaction. The concurrent validity of this subscale with the Oxford Happiness Questionnaire was 0.30 (Argyle et al., 1989). Cronbach’s alpha reliability was 0.87. In our study, Cronbach’s alpha was 0.89. The test was standardized with a sample of 1317 people, with a mean score of 32.76 (*SD* = 9.25).
(4) “Escala de Satisfacción Familiar por Adjetivos” (ESFA; in English, the Family Satisfaction by Adjectives Scale; Barraca Mairal and Yarto-López Elizalde, 2003). This instrument provides information about people’s cognitive and affective appraisal of the verbal and physical interactions with their family members. It attempts to evoke affective responses to the stem statement “When I am at home with my family, I feel rather …” People choose from 27 pairs of antonym adjectives (i.e., happy/unhappy, criticized/supported, etc.) the adjective (i.e., happy or unhappy) and score, ranging from 1 (*A little*) to 6 (*Completely*), that best describe their feelings. Scores range from a minimum of 27 to a maximum of 162, with higher scores indicating higher levels of family satisfaction. In general, scores above the mean indicate family satisfaction (scores exceeding percentile 70 are considered high, and scores below percentile 30 are considered low). Cronbach’s alpha internal consistency was 0.97, and reliability was 0.96. Its criterion validity with the Family Satisfaction Scale (Olson and Wilson, 1982) was 0.65, and with the Family Satisfaction Scale (Carver and Jones, 1992), it was 0.78. It has been adapted in other Spanish-speaking countries, like Peru, where it obtained Cronbach alpha reliabilities of 0.97 (Altamirano, 2008) and 0.95 (Chapi Mori, 2012). In our study, Cronbach’s alpha internal consistency was 0.93. The standardization of this test was conducted with 274 subjects from the general population. The mean score of the normative sample was 123.05 (*SD* = 24.59).

### Statistical Analysis

Through Student’s *t*-test, the mean scores of our sample on state-anxiety, material well-being, job satisfaction, and family satisfaction were compared with the mean scores of the normative samples of the above-mentioned standardized instruments. This analysis was complemented with the effect size using Cohen’s *d* to avoid a Type II error (failing to detect actual differences because of the differences in the sample size). Subsequently, the relation between these variables was analyzed with Pearson correlation coefficients. Lastly, we carried out a generalized linear model using univariate factorial analysis of variance, concurrently relating each measure of the dimensions of subjective psychological well-being to the sociodemographic variables of the sample. This analysis was complemented by the effect size using η*^2^*. Analyses were performed with the statistical program SPSS version 21.0.

## Results

The participants of our study scored higher on anxiety and lower on material well-being than the normative sample (see **Table 1**). However, these differences did not reach statistical significance, suggesting that the levels of anxiety and material well-being experienced by families with blind children are not very different from those of the general population. We note that the effect size of material well-being was high (*d* = 0.72), which could indicate that this lack of significance is due to sample size. However, the mean scores on job satisfaction and family satisfaction of parents with blind children were higher than the mean scores of the general population. Although these differences were not statistically significant, the effect size was moderate in both analyses (*d* = 0.39, and *d* = 0.42, respectively, for job and family satisfaction). In addition, as can be observed in **Table 1**, there were no statistically significant differences as a function of gender in material well-being, job satisfaction, state-anxiety, or family satisfaction (*p* > 0.050).

**Table 1:** Descriptive statistics and comparison of the variables material well-being, job satisfaction, and family satisfaction with normative samples.

Variable	Parents of blind children		Normatie samples		Student *t*-test	*gl*	*p*	*d* (effect size)
						
	*M*	*SD*	*M*	*SD*
Material well-being	27.21	9.85	33.48	8.90	–0.63	60	0.530	–0.70
Male	27.50	10.28	33.11	8.72	–0.53	27	0.596	–0.58
Female	26.96	9.63	33.72	9.01	–0.69	32	0.495	–0.72
Job satisfaction	35.55	6.81	32.76	9.25	0.40	48	0.687	0.39
Male	36.82	5.15	34.46	8.76	0.44	22	0.658	0.32
Female	34.42	7.93	31.65	9.47	0.34	25	0.734	0.31
State-anxiety	24.96	11.71	21.92	11.23	0.36	60	0.717	0.26
Male	23.21	11.91	20.54	10.56	0.22	27	0.827	0.23
Female	26.50	11.50	23.30	11.93	0.51	31	0.614	0.27
Family satisfaction	132.23	18.18	123.50	24.59	0.47	60	0.636	0.42
Male	134.50	17.11	123.97	22.48	0.60	27	0.551	0.52
Female	130.30	19.09	122.26	26.30	0.41	32	0.681	0.34


*State-anxiety was measured with the State subscale of the State-Trait Anxiety Inventory (Spielberger and Sydeman, 1994). Material and Job Satisfaction were subscales of the Scale of Psychological Well-Being Scale (Sánchez-Cánovas, 2007) and family satisfaction was measured with the Family Satisfaction by Adjectives Scale (Barraca Mairal and Yarto-López Elizalde, 2003).*

Correlational analysis revealed negative correlations between anxiety and material well-being (*r* = –0.62, *p <* 0.001), between anxiety and family satisfaction (*r* = –0.57, *p <* 0.001), and between anxiety and job satisfaction (*r* = –0.43, *p* = 0.008). That is, higher levels of material well-being, job satisfaction, and family satisfaction correlated with lower levels of anxiety in the parents of blind children.

We also observed a statistically significant positive correlation between job satisfaction and material well-being (*r* = 0.40, *p* = 0.004) and between material well-being and family satisfaction (*r* = 0.41, *p* = 0.001). This indicates that higher levels of material well-being are associated with higher levels of job satisfaction and family satisfaction. Or, contrariwise, a low level of material well-being indicates lower levels of job satisfaction and family satisfaction. No statistically significant correlation was found between family satisfaction and job satisfaction (*r* = 0.21, *p* = 0.204).

Lastly, **Table 2** shows the results of the univariate factorial analysis relating each dimension of subjective psychological well-being to the sociodemographic variables of the sample (parents’ sex, parents’ age, type of marriage, parents’ technical qualification, work wages, child’s visual level). As can be observed, only the variables “type of marriage” and “work wages” had an effect on the component of material well-being in these parents of visually impaired children. In contrast, no effects were found for the rest of sociodemographic variables or for their interaction.

**Table 2:** General linear unifactorial model to assess the effect of the sociodemographic variables on material well-being, job satisfaction, state-anxiety, and family satisfaction.

Variable	Material well-being				Job satisfaction				State-anxiety				Family satisfaction			
				
	*F*	*df*	*p*	η^2^	*F*	*df*	*P*	η^2^	*F*	*df*	*p*	η^2^	*F*	*df*	*p*	η*^2^*
Parents’ sex	0.13	1	0.719	0.00	0.01	1	0.920	0.00	0.08	1	0.774	0.00	0.08	1	0.773	0.00
Parents’ age	0.84	1	0.369	0.03	0.39	1	0.536	0.02	0.02	1	0.874	0.00	3.82	1	0.062	0.13
Type of marriage	6.73	1	0.016	0.21	0.08	1	0.775	0.00	0.61	1	0.441	0.02	3.00	1	0.096	0.11
Parents’ technical qualification	0.83	1	0.371	0.03	0.20	1	0.659	0.01	0.49	1	0.490	0.02	2.06	1	0.164	0.07
Work wages	4.09	1	0.050	0.14	3.12	1	0.094	0.14	0.09	1	0.767	0.00	0.41	1	0.524	0.01
Child’s visual level	0.46	2	0.631	0.03	1.38	2	0.276	0.13	1.15	2	0.333	0.09	0.16	2	0.846	0.01
				
Interactions	No significant interaction *p* < 0.050				No significant interaction *p* < 0.050				No significant interaction *p* < 0.050				No significant interaction *p* < 0.050			


## Discussion

As expected, when comparing the scores on state-anxiety of parents of blind children with those of the general population, we found higher anxiety scores in our sample. These differences were not statistically significant, but the effect size was moderate, similar to the data found by Femenías and Sánchez (2003) in an investigation of parents of disabled children. Authors like Dykens (2005) and Sola-Carmona et al. (2013) have reported that these families do not present generalized anxiety, and that the anxious response to having a disabled child is not homogeneous, suggesting that this variability is similar to that of the general population.

However, as predicted in the hypothesis, these families’ material well-being is lower than that of the general population, although the differences are non-significant (with a moderate effect size). In previous scientific publications, there was no clear evidence of how the socioeconomic status of families with disabled children may affect the parents’ anxiety. Authors like Friedrich and Friedrich (1981) and Dunst et al. (1986) and concluded that the two variables are related, but Byrne and Cunningham (1985) stated that there was no relation. Mayo Pals (2011), in a study with blind children’s families, defended that families with a higher socioeconomic status present lower rates of illness and mortality. In contemporary society, the salary earned and the material goods one possesses are very important, as noted by Garaigordobil et al. (2009), who reported the influence of material well-being on Spanish families’ subjective well-being. Other authors also confirmed that, besides the psychological impact of a child’s disability on the parents (increasing their levels of stress, anxiety, and depression), these families also suffer financial losses (Lukemeyer et al., 2000; Park et al., 2002; Bumbalo et al., 2005; Emerson et al., 2006; Msall et al., 2007; Bourke-Taylor et al., 2011).

In Spain, interventions are being implemented to favor of the collective of people with disabilities through actions like the Act of Social Integration of Disabled People (Law 13/1982, 1982) and the Act of Promotion of Personal Autonomy and Care of Dependent People (Law 39/2006, 2006), developing school integration and services for treatment/rehabilitation. This has led to a greater level of dignity and quality of life for this collective. In the specific case of blind people, the ONCE has formed a series of specialized services favoring their full integration. But despite all this, families with visually impaired children perceive that their economic income is lower and that they have fewer possessions than the normative sample. They think that they do not have enough resources to meet their children’s needs, as they must sometimes seek supplementary private resources. Authors like Badia Corbella (2002) have defended that counseling and economic aid for families with a disabled child are the most important factors to reduce family anxiety.

Although there were no statistically significant differences in the sample of parents with visually impaired children, it was found that job satisfaction was higher than in the standardized population (with a large effect size). This result is congruent with previous studies that have observed that the father’s work-focused role is beneficial for his contact with his blind child (Calvo and González, 2004; Durán Estrada, 2011). Coinciding with the generalization of women’s integration in the workforce, other authors have also observed that paid work can be considered a gratifying protective factor that may improve the well-being of mothers of disabled children (Einam and Cuskelly, 2002; Eisenhower and Blacher, 2006; Olsson and Hwang, 2006; Bourke-Taylor et al., 2010). Although in our work, we found no statistically significant differences between men and women with regard to job satisfaction, other authors like Chapi Mori (2012) found higher job satisfaction in women than in men, and attributed it to personal achievement, recognition of their work, their contribution to society, greater social participation, and a feeling of independence. In general, our results suggest that holding a job in these families with blind children allow them to recharge their energy and refresh their emotional relations when caring for a loved one like a child. We can observe a dichotomy: on the one hand, their child, who is the most important person in the world for them but, on the other hand, temporal distancing is also positively valued. Therefore, it would be appropriate for the authorities to facilitate access to or permanence in a job by increasing schedule flexibility, so that both parents can reconcile the care of their blind child and their work activity.

Our results also show that family satisfaction in parents of visually impaired children is higher than in the normative population, although there were no statistically significant differences (with a moderate effect size). This result was unexpected, taking into account previous studies in which it has been suggested that the birth or the diagnosis of a child with a disability necessarily implies family reorganization (Badia Corbella, 2002; Femenías and Sánchez, 2003). In a study with five disabled children’s families, Femenías and Sánchez (2003) obtained a lower score on family satisfaction than the control group formed by parents who did not have disabled children. Nevertheless our results suggest that this situation is currently changing and that these families display greater satisfaction, and the relations among their members are more positive. This may be due to the fact that having a blind child frequently causes the family to focus on the child, as if he or she granted a new meaning to life, in spite of the exhaustion involved in the child’s care (Sola-Carmona et al., 2013).

Finally, regarding our second objective, our results have revealed that the four scores (family satisfaction, state-anxiety, material well-being, and job satisfaction) used to measure the three components of subjective psychological well-being in our sample of parents of blind children correlated with each other, and the correlations were statistically significant (except for the relation between job satisfaction and family satisfaction). It is important to note that the correlations between state-anxiety and the remaining variables were negative, indicating that higher levels of material well-being, job satisfaction, and family satisfaction are associated with lower levels of anxiety. In previous studies with another type of population (Chapi Mori, 2012), it was also observed that higher levels of anxiety correlated with lower family satisfaction. On the whole, these results confirm one of the demands frequently made by families with blind children. They also reveal that providing these families with more economic resources would have a positive impact on their subjective psychological well-being, decreasing their state-anxiety and increasing their life satisfaction.

In addition, Sánchez-Cánovas (2007) described a moderate relation between material well-being and job satisfaction. However, our findings differ from the results of Sánchez López and Quiroga Estévez (1995), who had observed a positive and significant relation between job satisfaction and family satisfaction, and those of Femenías and Sánchez (2003), who found no correlation between situational anxiety and family satisfaction.

Among the limitations of this investigation, we note the reduced sample size, as not all the parents contacted consented to participate in the study. With regard to the selected sample, not all the blind children had the same degree of disability, and the comparison with the control group was done using the same group as the population chosen to validate the questionnaires employed. Moreover, despite the anonymity of the questionnaires, social desirability may have distorted some answers.

In spite of these limitations, the results of this study are very relevant to further our knowledge of the variables that can help to improve the psychological well-being of the parents of blind children.

A blind child’s parents are essential agents for his/her good development. Nowadays, the experiences of parents in this situation are changing. The parents express discomfort but they also acknowledge what raising an impaired child means for them. Therefore, this research was designed using many daily life aspects, such as family relations, work, and the economy of families with blind children.

Although these families feel comfortable, they also have a shortage of financial resources resulting from the expense incurred by treatments, trips, medication, and support personnel, as well as the uncertainty about the possible lifelong needs of the impaired child. The government should become more involved because, in spite of the update of Acts in favor of disabilities and the escalation of public services, it does not seem that enough is being done. This aspect requires further study so that this collective does not lose its quality of life and, ultimately, become marginalized. Such a situation would clash with the values of social integration and inclusion encouraged in our society, and which may be truncated.

## Author Contributions

JS-C: contribution to the conception and design of the work; the acquisition and interpretation of data for the work, revising it critically for important intellectual content and final approval of the version to be published. RL-L, MD, and DP-G: contribution to the conception and design of the work; the interpretation of data for the work, revising it critically for important intellectual content, and final approval of the version to be published. JA-P: contribution to the design of the work; the analysis of data for the work, revising it critically for important intellectual content and final approval of the version to be published. All authors are accepting and agreeing that the work is original; any methods and data presented are described accurately and honestly; any relevant interests have been disclosed.

## Conflict of Interest Statement

The authors declare that the research was conducted in the absence of any commercial or financial relationships that could be construed as a potential conflict of interest.
